# Prototype of a Spring-Loaded Module for Axillary Crutches

**DOI:** 10.3390/s25020296

**Published:** 2025-01-07

**Authors:** Dalia Danely Méndez-Gómez, Arturo Minor-Martínez, Salvador Montoya-Alvarez, Fernando Pérez-Escamirosa, Jessica Cantillo-Negrete

**Affiliations:** 1 Bioelectronics Section, Department of Electrical Engineering, Center for Research and Advanced Studies of the National Polytechnic Institute (CINVESTAV–IPN), Av. IPN 2508, Col. San Pedro Zacatenco, Mexico City 07360, Mexico; aminor@cinvestav.com; 2 Institute of Applied Sciences and Technology (ICAT), National Autonomous University of Mexico (UNAM), Circuito Exterior S/N, Ciudad Universitaria, Coyoacán, Mexico City 04510, Mexico; salvador.montoya@icat.unam.mx (S.M.-A.); fernando.perez@icat.unam.mx (F.P.-E.); 3 Division of Research in Clinical Neuroscience, Instituto Nacional de Rehabilitación Luis Guillermo Ibarra, Mexico City 14389, Mexico; jcantillo@inr.gob.mx

**Keywords:** ground reaction force (GRF), gait, kinetic parameter, spring-loaded, axillary crutches

## Abstract

Axillary crutches assist people with lower limb injuries but can lead to upper limb strain with extended use. Spring-loaded crutches offer a potential solution, yet they are rarely tested in clinical settings. This study developed spring-loaded crutches with an integrated force-measuring system to analyze gait dynamics. Three prototypes, each with different spring constants (k), were tested. To measure ground reaction force (GRF), a Nylamid cover was around the crutch stem. Two participants with different weights completed a 15-m route using both the designed spring-loaded and standard crutches. Findings showed that spring-loaded crutches increased mean GFR and impulse, with the prototype matched to the user’s weight yielding the best results. The study’s findings suggest that when properly adjusted to the user’s weight, spring-loaded crutches can offer significant improvements in gait, which may have important implications for the design of mobility assistive devices.

## 1. Introduction

Walking is a fundamental activity essential for human independence and mobility, yet it can be significantly hindered by factors such as age, illness, or physical injury. Axillary crutches, one of the primary assistive devices developed to support individuals with mobility challenges, help users regain movement during rehabilitation. However, prolonged use of conventional axillary crutches often leads to adverse effects on the upper body, including potential damage to the radial and ulnar nerves due to the direct pressure applied to the armpit region [1,2,3]. This nerve compression and strain can cause numbness, tingling, and even long-term nerve damage in some cases, particularly if crutches are not used correctly or for extended periods, redistributing body weight from the legs to the upper body, primarily engaging the armpits, arms, and shoulders. This shift in biomechanics requires significant upper-body strength and endurance, leading to increased physical effort from the user [4]. Consequently, crutch users expend more energy and often face discomfort in areas such as the shoulders, wrists, and hands. The repetitive strain on these joints increases the risk of joint pain and injuries, potentially resulting in complications like embolisms and thrombosis from reduced circulation [5,6,7]. This strain on the upper body highlights the need for an improved crutch design that minimizes these risks.

Although studies exist about the implementation of springs in axillary crutches, these focus on using a single spring without the need for adaptation according to the users’ anthropometric characteristics [8,9,10,11].

A spring-loaded system incorporated into the crutch stem was proposed to reduce the trauma caused by axillary crutches, improve efficiency, and reduce complications associated with prolonged use. A measurement system was also designed to record the ground reaction force (GRF) generated by a person walking with axillary crutches to measure the effects of the spring-loaded crutches compared to the conventional crutches.

This study provides information on the effectiveness of crutches with spring-loaded systems in reducing the adverse effects of prolonged conventional crutches. By directly comparing spring-loaded crutches with conventional crutches, potential improvements in assistive device design may be identified, which could benefit crutch users in the long term. The analysis of mechanical efficiency and the influence of the elastic constant will also help us better understand how material properties can optimize the performance of crutches, providing a basis for developments in the engineering of mobility assistive devices.

## 2. Materials and Methods

This work details the design and construction of a spring-loaded ground reaction force (GRF) measurement system for axillary crutches, enabling the measurement of compression forces exerted during walking. To evaluate gait efficiency, two pairs of crutches were tested: one unmodified pair, serving traditional axillary crutches, and a modified pair with integrated sliding mechanics. These mechanics allowed for testing three variations, each equipped with a helical compression spring of a different elastic constant (k). The modified crutches aim to enhance comfort and stability during movement, potentially reducing user fatigue. Descriptions of the design, testing setup, and results are presented below, highlighting the development of the spring-loaded system and its impact on gait dynamics.

### 2.1. Spring-Loaded Stem Design and GRF Measurement System

A specialized spring-loaded system for orthopedic use was designed and built, which was attached to the tubular member of axillary crutches. The system is configured to be inserted and secured within the tubular member using clamps.

The standard tubular member (5A) extends from its junction with the crutches and consists of a cylindrical body of specific diameter and length. The system includes a cushioning chamber (a), formed by a tubular body that, in turn, has a fixed lower cover (b), a mobile lower cover (c), and a mobile upper cover (d); inside, there is a helical spring of constant weight (e); the mobile upper cover has a screw (f), which is coupled through a threaded cylindrical plate (g) on it. The lower end of the screw rests on the cylindrical plate located inside the cushioning chamber; the position of the plate keeps the screw in a stable and punctual position when variable pressure is exerted on the spring.

It has a vertical stem (h) that passes through the fixed lower cover and joins the mobile lower plate in contact with the spring. The lower end of the stem is in contact with the tip (i) belonging to the body of the crutch.

**Figure 1:**
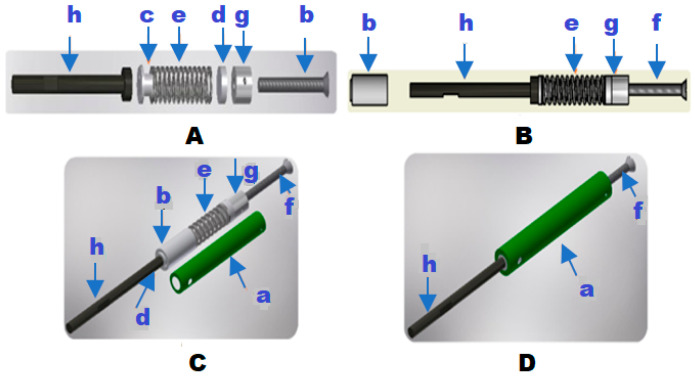
Sequential mechanical assembly of the spring-loaded module. (**A**,**B**) are lateral views of the module, (**C**) shows an isometric view, and (**D**) presents a fully assembled module with its final adjustment.

Figure 1 illustrates the mechanical assembly of the spring-loaded module. (A) and (B) provide lateral views, detailing the module’s individual components and overall structure, while (C) presents an isometric view, offering a more comprehensive perspective. This figure highlights the internal composition of the spring-loaded module and its final adjustment, as shown in (D).

To compare the gait between crutches with and without the proposed spring-loaded system, a measurement system was designed and built to record the ground reaction force (GRF). The system comprises an FX1901-00001-200L load cell whose internal structure is based on a Wheatstone bridge that allows precise detection of mechanical deformations. This sensor allowed us to measure GRFs in the range of 10 to 200 lbf (equivalent to an approximate range of 44 N to 889 N) with a sensitivity of $37.8\;\frac{mV}{V}$.

The load cell was powered with a voltage of 5 V, the output voltage at its maximum load was measured, and a voltage of $V_{LoadCell}\left(889\;N\right)=170\;mV$ was obtained. This load cell signal was conditioned using an INA129P instrumentation amplifier to amplify the signal so that $V_{out}\left(889\;N\right)=5\;V$. The signal also presented an offset error of 87.8 mV, which was corrected with an LM358 operational amplifier in a differential configuration. An ATMEGA328P microcontroller digitizes the conditioned signal at a sampling rate of 100 Hz.

A hydraulic press was used to exert known forces on the sensor and obtain the total system reading. Forces were taken over the entire measurement range at intervals of 49.05 N. For each force value, an average of 10 readings was taken. The data obtained were fitted to a straight line using the least squares method, yielding the coefficients of a first-degree function expressed as $f\left(x\right)=ax+b$. Based on these coefficients, the system’s output voltage was established as $V\left(GRF\right)=\left(4.8863\;\frac{mV}{N}\right)\left(GRF\right)-37.9620\;mV$, correlating force with voltage response.

**Figure 2:**
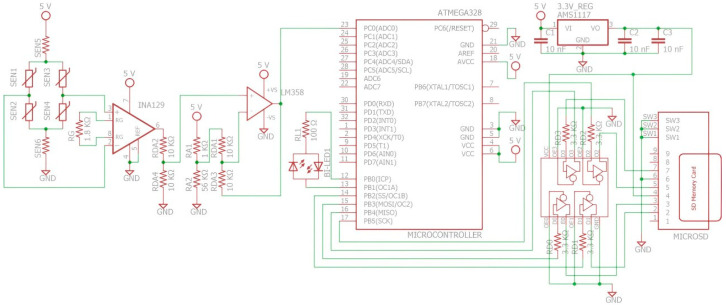
Sensor conditioning electronic schematic: Red symbols represent electrical components, green lines represent wire connections, and green dots indicate electrical connection nodes.

This calibration curve was programmed in the microcontroller to calculate the GRF exerted during walking from the voltage read at the ADC input. The measured GRF values are stored in a microSD memory. Figure 2 shows the schematic diagram of the measurement system.

**Figure 3:**
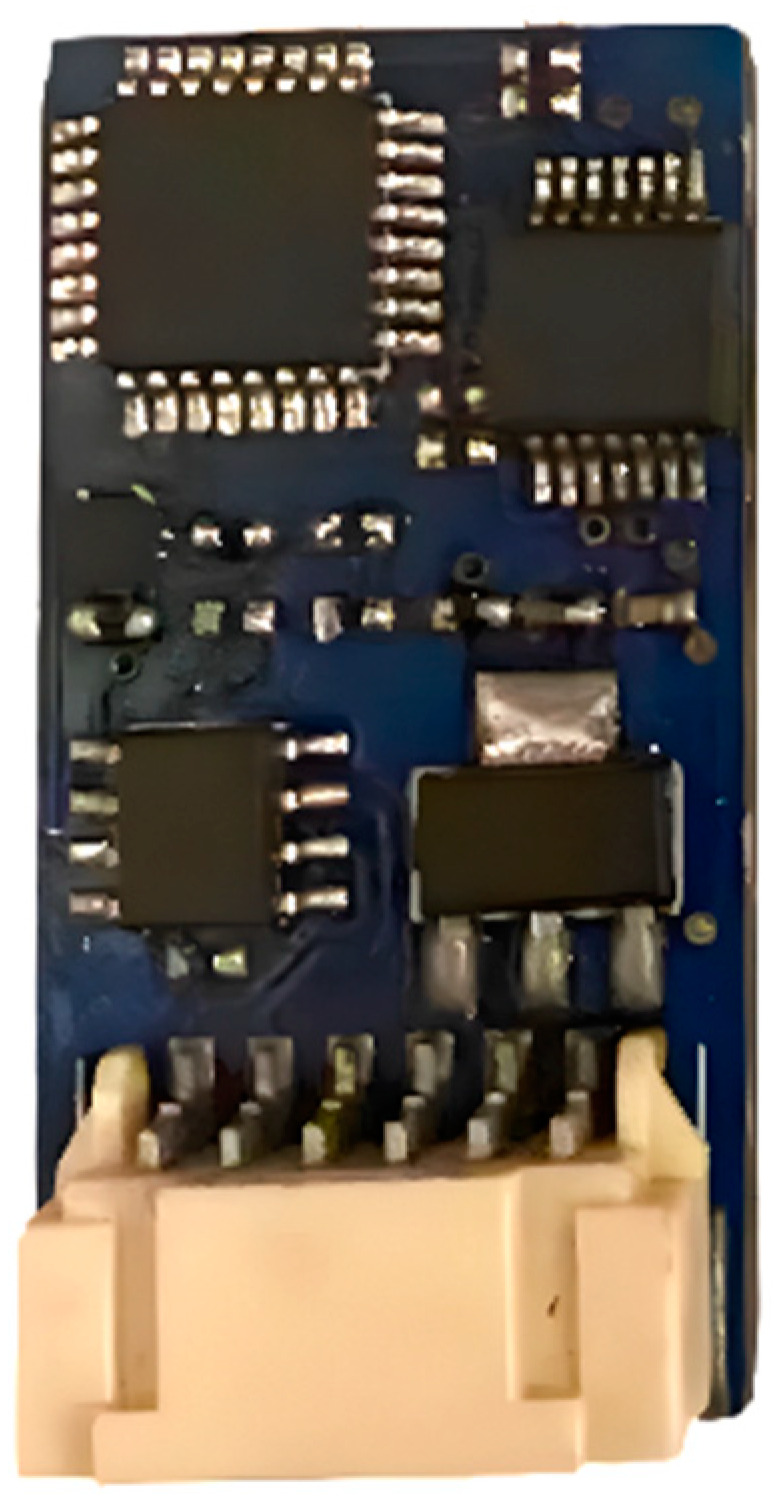
Front view of acquisition system circuit board.

**Figure 4:**
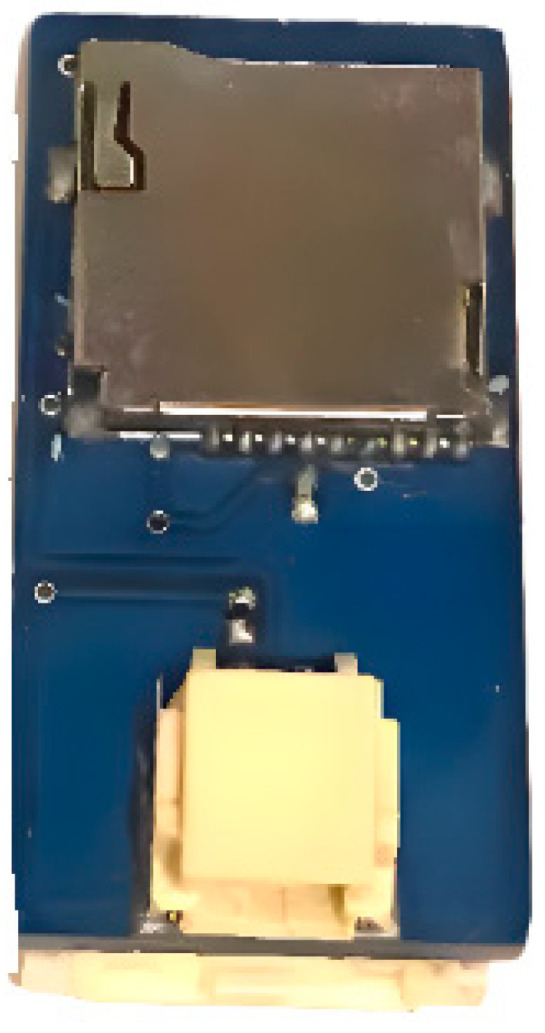
Rear view of the acquisition system circuit board.

Figure 3 and Figure 4 illustrate the recording system on a printed circuit board. Figure 3 shows the front view of the acquisition board, where the FX1901 sensor is connected at the bottom through a dedicated connector. Meanwhile, Figure 4 presents the rear view of the acquisition board, highlighting the location of the microSD reader, which is used to store the force data recorded during the tests.

**Figure 5:**
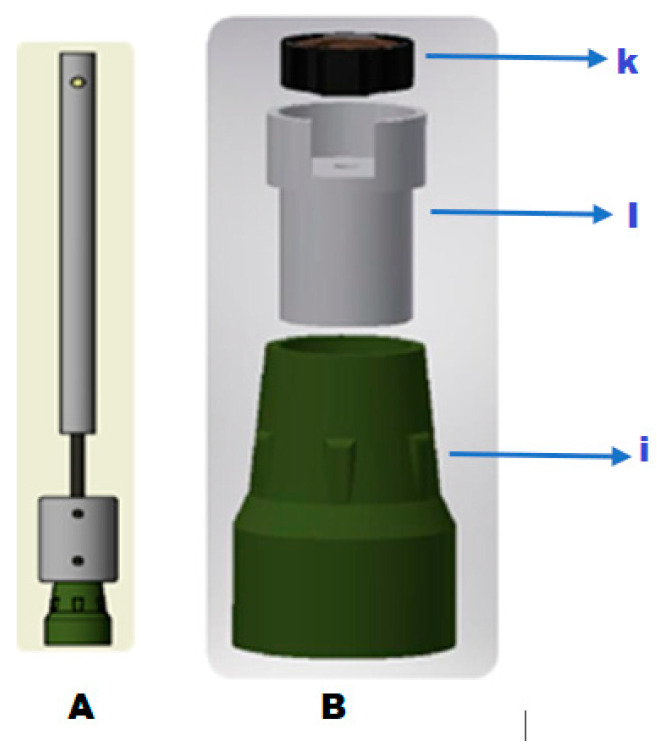
Adjusting the spring module to the tubular base of the crutch, where: (**A**): Side view of the crutch shaft, internally housing the spring-loaded module, extending down to the crutch tip. (**B**): Assembly of the force sensor onto the cylindrical clamp, integrated with the crutch tip.

Figure 5 shows (A) the spring-loaded module coupled to the standard crutches’ tubular member. (B) At its lower end, the FX1901 sensor is attached to the crutches’ tip (i), which is joined by a cylindrical piece (l) according to the dimensions of the sensor (k).

**Figure 6:**
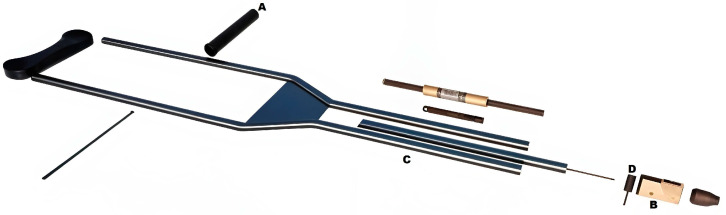
Design of the crutch with a spring-loaded system and its components (**A**) hand grip, (**B**) acquisition system, (**C**) spring-loaded system, (**D**) FX1901L sensor coupling.

Figure 6 illustrates the conventional crutch and both systems’ incorporation into the axillary crutches.

The developed device costs MXN 1,000, which is comparable to the conventional device, typically priced at MXN 700, so the cost is not prohibitive to users.

### 2.2. Measured Variables

This study reports two variables: the average GRF and the average impulse. Impulse is mathematically defined as the area under the curve of the rising edge of the GRF signal, and mechanically, it is the energy released by the spring to affect a step. The reported impulse was obtained from the average of each course’s steps for each pair of crutches.

The average GRF is the average magnitude of the recorded force signal. The GRF signal is composed of two half-cycles and has a shape similar to a square wave.

Semi-cycle with positive magnitude: This represents the phase in which the patient relies on crutches. During this phase, the crutches support the patient’s weight, which causes pressure on the sensor that measures the GRF. The positive magnitude indicates the force exerted by the patient’s weight.

Semi-cycle with zero magnitudes: This represents the swing phase of the crutches when the patient’s uninjured leg supports the body’s weight. In this phase, the sensor has no pressure because the crutches are not in contact with the ground, resulting in a GRF reading of zero.

The recorded GRFs went through a windowing process to evaluate the change in characteristics of the spring-loaded system during walking concerning the standard crutch. This process consists of segmenting the signal into parts corresponding to each step.

A proof of concept was carried out to analyze the spring-loaded module effects on walking.

### 2.3. Proof of Concept

Two pairs of crutches were used in this study. One pair was a standard, unaltered axillary crutch, serving as the control. In comparison, the other pair featured a modified, spring-loaded system with interchangeable springs. Each spring had a unique elastic constant adjustment for users of different weights. This spring-loaded design was incorporated to assess its impact on user comfort and ground reaction force measurement. Both crutch pairs integrated the GRF measurement system for comprehensive data analysis.

This study was conducted at the Center for Research and Advanced Studies of the National Polytechnic Institute. Three helicoidal compression springs were tested to accommodate different weight categories. The first spring was designed for users weighing over 80 kg, the second for users between 70 and 75 kg, and the third for lighter individuals weighing 35–40 kg. Each spring’s elastic constant varied, allowing researchers to compare the effect of different spring properties on GRF measurements and user experience. These specifications are detailed in Table 1, illustrating the spring’s diameters, lengths, and maximum load capacities. The structured variation in spring characteristics provides insights into the optimal spring design for enhancing crutch-assisted mobility, aiming to improve the functionality and comfort of standard axillary crutches for various body weights.

The test consisted of traveling 15 m in a straight line with each pair of crutches. The start and end positions of the route to be traveled were marked to standardize the route. Test subjects simulated a lower trunk disability by elevating their right leg during the test. For each pair of crutches analyzed, three repetitions were performed for each type of spring for each test subject.

The participants were asked to exert force in the direction of the ground with the crutches for 2 s of sustained force of 32 N (3.5 kg) to activate the recording system and begin the test.

After all the trials were completed, the resulting GRF signal of each trial was extracted from the measurement system’s microSD memory. The GRF signal was processed in MATLAB R2021a software to obtain the mean GRF and impulse.

## 3. Results

Two healthy volunteers participated in the proof of concept, the first subject weighing 73 and the second weighing 83 Kg. Both participants had previous experience with axillary crutches and simulated a lower limb fracture in the right leg. The 73 Kg participant completed all the requested trials. However, the 83 Kg participant’s trial with prototype three was suspended because he reported discomfort during the test.

**Figure 7:**
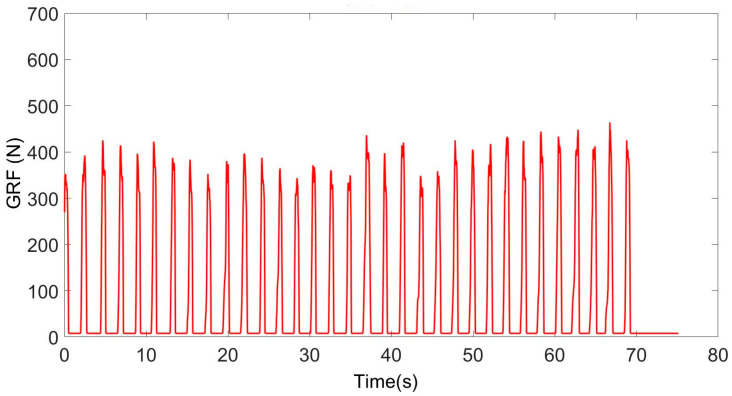
Signal obtained from the distance taken while walking with crutches.

Figure 7 shows the GRF signal obtained from the 15 m distance taken while walking with crutches.

**Figure 8:**
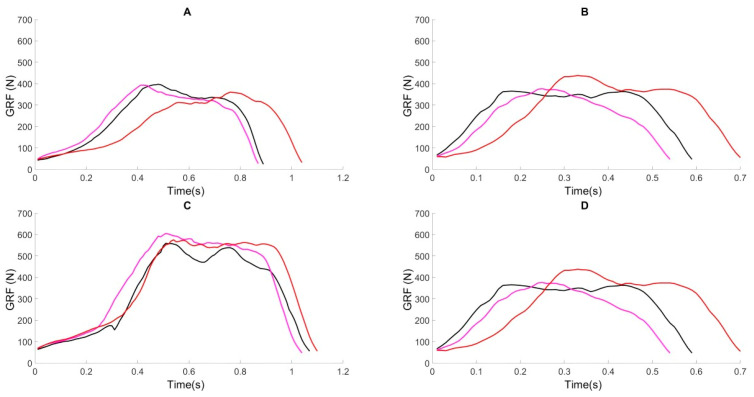
Individual step graphs for participant 1 under four conditions: (**A**) using standard crutches, (**B**) using a spring-loaded crutch with spring 1, (**C**) using a spring-loaded crutch with spring 2, and (**D**) using a spring-loaded crutch with spring 3. In each condition, the red line represents the ground reaction force (GRF) during the first trial, the magenta line represents the GRF during the second trial, and the black line represents the GRF during the third trial.

Figure 8 shows a windowed step of the 73 Kg participant in the three repetitions using the (A) standard crutch, (B) spring-loaded crutch with spring 1, (C) spring-loaded crutch with spring 2, and (D) spring-loaded crutch with spring 3. These graphs allow visual and quantitative comparison of each step, helping to identify gait patterns and differences.

**Figure 9:**
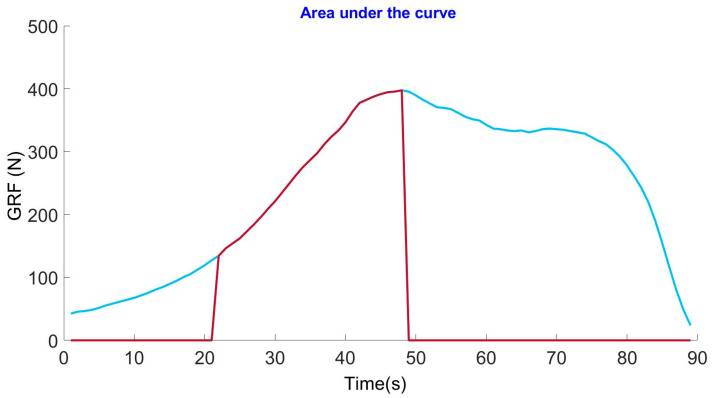
Area under the curve of a step, where the blue line represents the measured ground reaction force (GRF) signal, and the red line highlights the area under the curve that determines the impulse.

The mean impulse of each step during the travel was obtained to determine the spring’s effectiveness. The impulse representation is demonstrated graphically in Figure 9.

Table 2 reflects how the selection of the spring influences the mean impulse generated by each test subject when performing the trials with the spring-loaded crutches. For the 73 Kg subject, spring 2, designed for a weight of 70 to 75 Kg, produced the greatest impulse. Springs 1 and 3, although generating greater thrust than the standard crutch, were less effective than spring 2.

A similar pattern is observed in the 83 Kg subject. The ideal spring for the participant’s weight generated more impulse than the others, although the difference was not as marked as in the 73 Kg subject. Impulse and ground reaction force data could not be obtained with Spring 3, as the participant experienced significant discomfort with the crutch during the first trial. Upon investigation, it was found that the participant’s weight caused the spring to compress fully and quickly, resulting in the crutch pad applying painful pressure to the axilla. Consequently, testing with Spring 3 was discontinued for the 83 Kg participant.

Table 3 shows the mean GRF when the 83 Kg and 75 Kg participants occupied each spring. The GRF was greater than in any other case when the appropriate spring was used for the participant’s weight.

**Figure 10:**
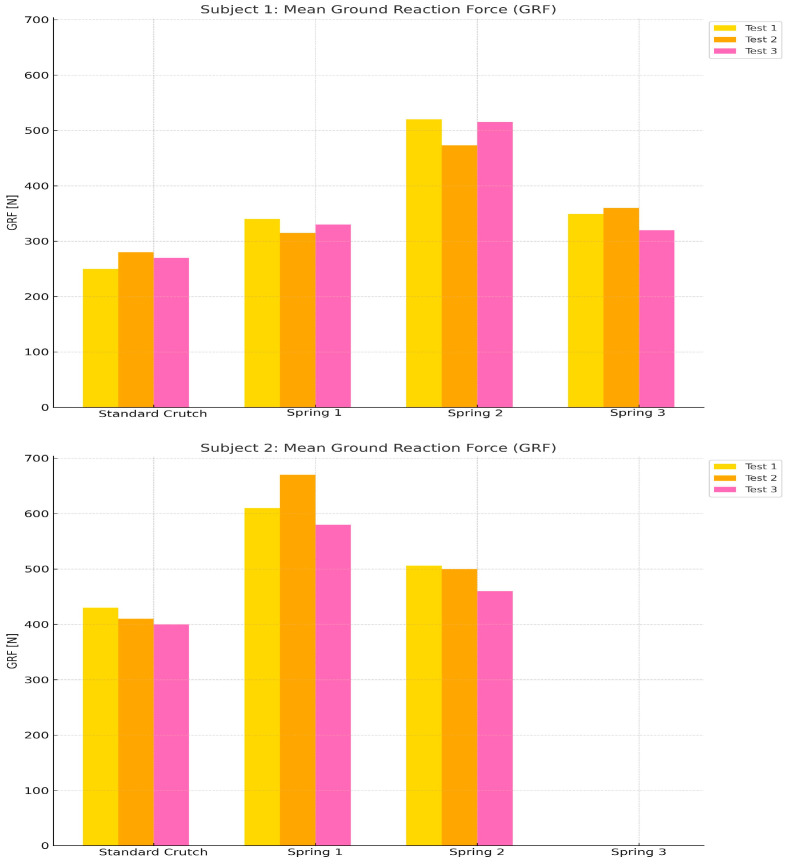
Bar graph of the mean GRF recorded by each subject under the four conditions.

**Figure 11:**
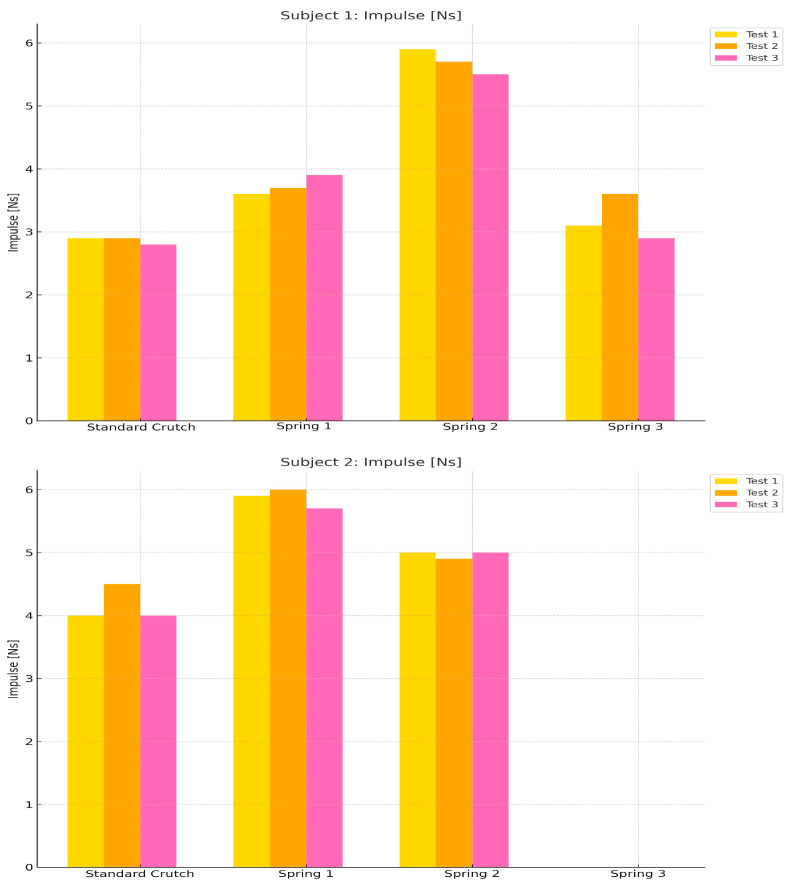
Bar graph of the impulse recorded by each subject under the four conditions.

Figure 10 and Figure 11 display the mean GRF and impulse data, respectively, in graphs for better interpretation, separately for each subject, to compare the mean GRFs under the different crutch systems used.

## 4. Discussion

This study highlights the benefits of using spring-loaded crutches optimized for individual body weight, offering substantial improvements in biomechanical efficiency and user comfort. Testing involved two participants using three different springs, with one specifically matched to each participant’s weight. Results showed that springs customized to the user’s weight significantly increased impulse—a measure of force over time—by enabling better energy absorption and return.

In this context, absorption and return describe the spring’s ability to handle mechanical energy throughout each step cycle. During the swing phase, as the user takes a step, the spring absorbs mechanical energy, temporarily storing it. This energy is then released during the stance phase, providing a boost that maintains high mechanical efficiency. This mechanism reduces the load on the shoulder compared to using non-spring-loaded crutches, allowing for a smoother and more comfortable experience by distributing effort more effectively. Overall, adjusting springs to match the user’s weight effectively enhances impulse, demonstrating how personalized spring selection can promote a more fluid and efficient movement experience, which is particularly valuable for long-term crutch users.

Additionally, ground reaction force (GRF) measurements revealed a consistent rise when participants used springs optimized for their weight. Enhanced GRF indicates improved load distribution and force absorption, allowing smoother, more controlled motion while reducing the risk of abrupt force shifts. This minimized force transition is crucial for long-term user comfort, as it helps reduce strain on muscles and joints. Figure 10 and Table 3 provide detailed data supporting these findings, illustrating the improvement in force management with customized springs.

The study’s findings underscore how weight-optimized springs allow for controlled compression and decompression, leading to better energy storage and release with each step. This efficiency relieves some of the impact forces that typically occur during walking, directly benefitting the user by reducing strain on their joints and muscles. In contrast, standard crutches lack this dynamic response, limiting their ability to cushion impact forces effectively. Well-matched springs not only improve stability and prevent discomfort from over-compression but also enhance the overall support provided with each step.

The research shows that spring-loaded crutches designed with weight-specific springs can improve comfort and biomechanical performance. Such spring selection offers a significant advancement over traditional crutch designs, emphasizing a more personalized approach to assistive devices. For individuals relying on crutches long-term, maximizing impulse and GRF with properly calibrated springs promotes ergonomic efficiency, reduces discomfort, and may lower the risk of injury from repetitive strain. By customizing crutches to the user’s weight range and walking mechanics, spring-loaded crutches emerge as a powerful solution for enhancing comfort, support, and movement efficiency. This study highlights the potential of personalized crutch design as a crucial factor in improving mobility, comfort, and long-term safety for users.

For the future development of this project, a structured approach is essential to refine and enhance the current system, targeting improvements that support its role in rehabilitation and post-surgical training. Key areas for improvement include optimizing the mechanical design, expanding user validation studies, integrating real-time feedback, and advancing data visualization.

- Mechanical Design Optimization

Enhancing the mechanical design of the crutches and spring-loaded-based system would allow for greater customization and adaptability for diverse user profiles. This could involve refining spring properties, exploring alternative materials, or using alloys to improve durability and mechanical responsiveness. Adjustable components would allow users to configure spring tension based on their weight, height, or specific physical needs. This modular approach could significantly increase the system’s versatility, ensuring it meets performance and ergonomic needs across a wide variety of users.

- Expanding User Validation Studies

Expanding the sample of test subjects is another step. A more comprehensive study could include participants with lower limb pathologies, as well as various weight categories and statures, to ensure the system’s effectiveness across a more representative sample. Longitudinal studies could also be conducted to assess system performance over time, investigating user fatigue and whether the device effectively reduces physical strain during prolonged use. These studies would provide valuable data on reliability, comfort, and the overall impact of the system on user recovery and rehabilitation, essential for validating the device before widespread implementation. This would also require implementing a research protocol to concisely explain to users the type of study being conducted and ensure adherence to ethical guidelines.

- Real-Time Feedback for Users

Integrating a real-time feedback system could significantly enhance the user experience by allowing continuous monitoring of exerted force. A haptic feedback mechanism, such as a portable vibration device or a visual indicator, could provide immediate feedback if the user’s force exceeds optimal ranges. This feature would help users maintain consistent force application, enhancing safety and reducing the risk of injury due to improper use.

- Advanced Visualization Interfaces and Simulation Models

The precise adaptation of the springs according to the user’s weight contributes to greater efficiency while riding, suggesting that a personalized configuration offers substantial benefits in terms of comfort and functionality.

The need to adjust the spring-loaded system for each specific user implies greater complexity in the design and execution of large-scale studies. This customization could become prohibitive, especially as the sample size increases, due to the cost and logistics of producing and fitting specific springs for each individual.

- Upper body analysis

The prolonged use of a conventional spring causes ailments to the upper body, which is not designed to deal with the whole body weight. While in this study, we analyzed changes in GRF and impulse, the effects of the system on the upper body are yet to be considered. The use of novel strain sensors, such as the one presented by Hong et al. [12], and portable electromyography systems is being considered to obtain a deeper understanding of the mechanical activity of upper body muscles during gait with spring-loaded crutches compared to traditional crutches.

- Ergonomic analysis

The developed spring-loaded crutch design was based on conventional axillary crutches since they are the most common type of crutches used. However, they are not the only type of crutches. To further reduce the strain on upper body muscles, an in-depth ergonomic analysis of the design of spring-loaded crutches is needed, along with guidelines to properly educate users on the proper use of these kinds of devices.

As we move towards studies with larger sample sizes, it will be crucial to find a balance between the system’s customization and the study’s feasibility. Solutions such as using adjustable springs or categorizing patients into broader weight ranges could be explored to reduce the number of variations needed without compromising the system’s effectiveness.

Although customization of the spring-loaded system shows clear benefits in terms of mechanical response and comfort, large-scale implementation presents challenges that must be carefully addressed to ensure the system’s feasibility and effectiveness in a broader clinical setting.

## 5. Conclusions

Adding springs adjusted to each user’s weight in spring-loaded crutches has proven to enhance efficiency and comfort, underscoring the value of a personalized configuration for those using axillary crutches. This individualized approach has the potential to optimize force distribution, minimize user fatigue, and improve overall functionality during movement, creating a smoother and more supportive experience.

However, the need to adjust the spring-loaded system to match each user’s specific weight and biomechanical profile introduces considerable complexity, especially when scaling up to studies with larger samples. As the sample size grows, so do the logistical and financial challenges associated with producing, calibrating, and fitting custom springs for each individual. This could limit the feasibility of personalized springs in broader studies of large-scale applications.

To address this, exploring alternative solutions like using adjustable springs or grouping participants by categories could strike a balance between system customization and practicality. Adjustable springs, which can be modified to suit a range of weights, may simplify logistics without sacrificing efficacy. Similarly, classifying participants into broader weight categories would reduce the number of required configurations, making the system more adaptable for large sample sizes. Both approaches could maintain system effectiveness while enhancing feasibility and scalability in studies of eventual real-world applications.

## 6. Patents

The proposed system has been approved and named “MODULO AMORTIGUADOR PARA USO ORTOPEDICO” with registration number 385531.

## Figures and Tables

**Table 1:** Characteristics of springs used.

Parameter	Spring 1	Spring 2	Spring 3
Spring type	Helicoidal compression spring	Helicoidal compression spring	Helicoidal compression spring
Outer diameter (De)	16 mm	15.50 mm	15 mm
Wire diameter (d)	3 mm	2.50 mm	2 mm
Length (L)	86 mm	88 mm	88 mm
Distance between coils (p)	5.33 mm	4.237 mm	4.42 mm
Number of active coils (N_a_)	15	19	19
Spring constant (k)	24.36 N/mm	9.269 N/mm	3.797 N/mm
Spring load (F_cth_)	516.25 N	325.51 N	174.64 N

**Table 2:** Mean impulse per subject reported in tests.

Impulse					
Test	Subject	Standard Crutch	Spring-Loaded Crutch		
			Spring 1 (Optimal for 80+ Kg)	Spring 2 (Optimal for 70–75 Kg)	Spring 3 (Optimal for 70–75 Kg)
1	1 (75 Kg)	2.9 Ns	3.6 Ns	5.9 Ns	3.1 Ns
	2 (83 Kg)	4.0 Ns	5.9 Ns	5 Ns	-
2	1 (75 Kg)	2.9 Ns	3.7 Ns	5.7 Ns	3.6 Ns
	2 (83 Kg)	4.5 Ns	6.0 Ns	4.9 Ns	-
3	1 (75 Kg)	2.8 Ns	3.9 Ns	5.5 Ns	2.9 Ns
	2 (83 Kg)	4.0 Ns	5.7 Ns	5.0 Ns	-

**Table 3:** Mean Ground Reaction Force generated by each subject in tests.

Mean Ground Reaction Force (GRF)					
Test	Subject	Standard Crutch	Spring-Loaded Crutch		
			Spring 1 (Optimal for 80+ Kg)	Spring 2 (Optimal for 70–75 Kg)	Spring 3 (Optimal for 70–75 Kg)
1	1 (75 Kg)	250 N	340 N	520 N	349 N
	2 (83 Kg)	430 N	610 N	506 N	-
2	1 (75 Kg)	280 N	315 N	473 N	360 N
	2 (83 Kg)	410 N	670 N	500 N	-
3	1 (75 Kg)	270 N	330 N	515 N	320 N
	2 (83 Kg)	400 N	580 N	460 N	-

## Data Availability

The raw data supporting the conclusions of this article will be made available by the authors upon request.
